# Universal health coverage in the framework of the 2030 global agenda for sustainable development: agreements and challenges

**DOI:** 10.7189/jogh.10.010316

**Published:** 2020-06

**Authors:** Armando Arredondo, Ana Lucía Recamán, Brenda Castrejón

**Affiliations:** 1 National Institute of Public Health, Cuernavaca, México; 2 La Salle University, Cuernavaca, México; 3 Center for Health System Research, Cuernavaca, México

The high-level summit on universal health coverage organized by the United Nations (UN) was recently held in the context for the revision of the 2030 Agenda for Sustainable Development (ASD). One of the main challenges discussed was the need to develop a global reference framework with key guidelines to ensure equal access to health services and achieve better universal coverage in both communicable and non-communicable diseases [1].

With a large and multidisciplinary participation of academics, politicians and health policy makers from around the world, based on discussion panels, a global framework agreement on universal health coverage was integrated. Such event took place at the UN headquarters in New York, as part of the debate of the General Assembly of the member countries. At the beginning of the summit, the General Secretary described the adoption of the agreement as a significant achievement that during the next decade will lead the fight against communicable diseases such as HIV / AIDS, tuberculosis and malaria, in addition to dealing in a relevant way of non-communicable diseases through solid and resilient primary care systems [1,2].

## RESULTS AND AGREEMENTS

Resulting as one of the most ambitious documents in history in the field of health at global level, the agreed points received the support of all UN member countries [1,2]. The urgency of acting now and guaranteeing access to health services with equal opportunities was recognized. It was agreed as a central axis to defend health as a human right, highlighting financial sustainability for effective universal coverage. For this purpose, the commitment of greater financial investment in health systems within each country was agreed, especially to strengthen primary care strategies linked to universal coverage. Specifically, the joint declaration proposes to make the exercise of the right to health to the fullest possible for all the inhabitants of the planet in a period no longer than 10 years [2,3].

During the development of this summit, one of the moments that caught most the attention was the message of the director of the WHO, highlighting what it represents and means the need to move towards new and better models of care with schemes where effective universal coverage is guaranteed [3]. That means, transit from normative universal coverage schemes based on the number of affiliated population; to effective universal coverage based on full attention with health services and medications required, particularly for groups of greater social vulnerability and financial deprotection in health. This will be of great relevance for the fulfillment of the goals of the 2030 agenda related to objective 3 [4]. Unless the course changes, globally it was noted that around 5000 million people will continue to lack universal access to essential health services in 2030 [5]. Within the framework of this summit, it was also discussed in different panels that the guarantee of effective universal coverage should be based on the implementation and monitoring of cost-effective interventions, even if it is not possible to have absolute cost-effectiveness thresholds. In this sense, it is also proposed that each country could define or adjust an appropriate approach to its context to make decisions about the most cost-effective interventions included in new universal coverage strategies [5].

Currently, the world spends about 10% of the global Gross Domestic Product in the health sector. This item is not efficient since, in many cases, it is intended for expensive infrastructure instead of promoting health and preventing diseases with primary care or with a more social than biomedical approach [3,5]. It requires a radical change and focus on promoting health instead of just curing diseases. We must invest much more in detection, promotion and prevention. On the other hand, it was emphasized that more than 80% of health needs can be met with quality primary services. In this context, all health ministers present and not present were asked to invest at least 1% of their Gross National Product in quality primary care [3]. Even the WHO director stressed that after all, health is a political decision and it is the politicians and their governments who have the duty and power to make that decision. According to WHO, this level of annual investment would save 60 million lives each year and extend life expectancy in 3.7 years by 2030 globally [5].

Concluded the summit, through different discussion panels among world leaders in health, in a joint declaration, they also agreed to reinforce achievements made to 2018, in order to achieve greater universal coverage in 2030, as the SDA marks. It was resumed as an urgent strategy to change course and make effective the right to health protection globally. All leaders, urgently propose to review and improve the implementation, monitoring and financing of new universal coverage schemes [3]. Greater investment was agreed to strengthen primary care interventions, in addition to improving financial protection and equal opportunities in access to health, both in communicable and non-communicable diseases. In a very important way and taking the axis of change of course as a thread, it was agreed to stop along the way to develop and analyze indicators of recent achievements in the field of universal coverage, strengthen successes and correct failures [3,5].

## CHALLENGES

From our perspective, all countries face challenges that can hardly be resolved to achieve the goals. Undoubtedly, the biggest challenge is to move from universal normative-raw coverage (it faces barriers in total access to services and medicines), towards effective universal coverage (it does not face any access barrier) [6,7]. This implies facing and solving historical challenges in terms of accessibility. It is necessary to confront and monitor the resistance of health systems: geographical resistance, financial resistance and cultural-organizational resistance. As the new coverage schemes decrease the effect of these 3 categories of access resistance, the power and access of users to health services will be significantly increased [7].

The challenges could be complicated by analyzing the political feasibility of the changes that are required to be implemented within each country. Moreover, if we take into account that global agreements of this type have a great weight from the perspective of international political economy systems, monitored by multilateral global agencies (United Nations, World Health Organization, International Monetary Fund, Regional Government Organizations, etc.). Indeed, the contrast between philosophy, agreements and guidelines from a global policy framework may be in accordance or in conflict with the social and economic policy systems within each country.

In this sense, the political feasibility of implementing the summit agreements and facing the challenges in each country, will depend on the levels of governance between the different social actors involved (local level, regional level and global level). The process can get more complicated and with low political feasibility in some countries; especially where health systems favor private practices in medical care, with benefits oriented to providers and with low regulation of the health services market. On the other hand, contexts favorable to the summit agreements will be those countries where health systems do not promote private practices and are rather patient-oriented, with collective benefits of users, mainly in terms of the population's health needs.

**Figure:**
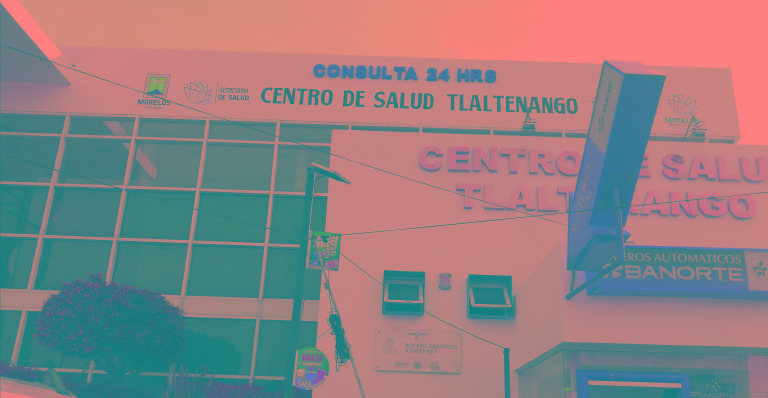
Photo: Health Care Center under universal coverage program in Mexico. From the collection of Dr A Arredondo (used with permission).

Returning to one of the proposals and agreements of the summit, in the sense of reviewing achievements, strengthening successes and correcting failures, the challenge of developing different monitoring indicators to generate inputs for decision making cannot be delayed. Although relevant points were certainly proposed and agreed upon for the development of a global agenda in the field of new and varied universal coverage schemes, unfortunately, the indicators that will measure the achievements in relation to objective 3, closely related to the goals of universal health coverage, were not addressed or proposed in a timely manner.

As part of the main challenges, we want to highlight that the universal coverage schemes in force in all countries face the daily challenges of the care model on which they are based. It will be difficult to improve the universal coverage schemes, if we continue with schemes based primarily on a biomedical model, with a curative approach, with high investment in complex technology medicine, low investment in preventive medicine and with low primary care; where also the social determinants are not considered as a relevant variable of the state of health [7].

Stopping along the way to strengthen successes and correct failures faces the challenge of having very specific evidence / indicators that were not considered in the aforementioned agreement. Precisely in this context and in a synthetic way, we want to highlight the challenge of reviewing and monitoring globally and within each country the scope [8]. For this purpose, we take as reference some indicators of achievement of universal coverage 13 years after its implementation for two chronic conditions in a middle-income country [7-10]. We propose that to make decisions for continuous improvement of coverage schemes on the road to 2030, each country could monitor the trend of its coverage and financial protection achievements at least for the top 10 health priorities. Such indicators can be differentiated in annual rate of normative-raw universal coverage vs effective universal coverage. Likewise, we suggest the challenge of monitoring financial protection indicators based on trends in the annual expenditure of patients' pocket by type of illness (**Table 1**).

**Table 1:** Some key indicators of effective universal coverage and financial protection for diabetes and hypertension in Mexico*

Indicator	Diabetes	Hypertension
Total coverage rate reached until 2015	60%	50%
Annual normative-crude coverage rate reached until 2015	35%	27%
Annual effective coverage rate reached until 2015	25%	23%
% of out-of-pocket expense in relation to total annual expenditure in 2005	49%	47%
% of out-of-pocket expense in relation to total annual expenditure in 2015	46%	43%

*Source: Developed by authors with data from [7,9,10].

## CONCLUSION

Facing the challenges to move from universal normative-raw coverage to effective universal coverage will only be possible with broad participation and joint alliances of all the actors involved: levels of government, health care professionals, decision makers in public policies, researchers, civil society organizations, service users, leaders of the legislative branch, community leaders and leaders of regional and global agencies. Only in this way will it be possible to design, promote and implement changes of substance and form quickly and effectively. We are in a historical moment where it is essential to move from universal coverage schemes based on a biomedical-curative care model with little primary care, towards schemes based on a bio-socio-medical-preventive model with multiple primary care interventions and with a preponderant role of Social Determinants of Health. Otherwise, little progress will be made on the goals of the ADS. In summary, the greater the degree of adjustment and changes in the coverage schemes that have been implemented until 2018 in all countries, the greater the scope of the goals by 2030.
